# SALL4 is a novel therapeutic target in intrahepatic cholangiocarcinoma

**DOI:** 10.18632/oncotarget.4862

**Published:** 2015-08-07

**Authors:** Gang Deng, Lei Zhu, Feizhou Huang, Wanpin Nie, Wei Huang, Hongbo Xu, Shaopeng Zheng, Zhongjie Yi, Tao Wan

**Affiliations:** ^1^ Department of Hepatobiliary and Pancreatic Surgery, the Third Xiangya Hospital of Central South University, Changsha, Hunan 410013, P.R. China

**Keywords:** intrahepatic cholangiocarcinoma, SALL4, proliferation, migration, invasion

## Abstract

Intrahepatic cholangiocarcinoma (ICC) is the most common and deadly disease of the biliary tree due to its poor prognosis. Sal-like protein 4 (SALL4), a stem cell marker, has been identified as a potential target for aggressive hepatocellular carcinoma (HCC). In our study, 175 ICC cases with an average age of 55 years were included, and 53% (93/175) were male. And 28 adjacent non-tumor tissues were also collected. The SALL4-positive immunoreactivity was detected in a total of 102 ICC cases (58%), whereas all 28 adjacent tissues showed negative staining. Univariate analysis, showed that the SALL4-positive ICC cases had significantly more frequent lymph nodal metastasis (*P* = 0.0460), vascular invasion (*P* < 0.0001), and nerve invasion (*P* < 0.0001). Furthermore, the strong SALL4-positive cases (*n* = 7, 5 months) had shorter overall survival, when compared to moderate SALL4-positive (*n* = 46, 9 months) or SALL4-negative cases (*n* = 73, 7 months), respectively. Our data also suggest that SALL4 may be involved in the regulation of epithelial-mesenchymal transition (EMT) in ICC. Those results for the first time indicate an oncogenic role of SALL4 in ICC. Therefore, SALL4 may serve as a promising therapeutic target for ICC.

## INTRODUCTION

Intrahepatic cholangiocarcinoma (ICC) is the most common neoplasm of the biliary tree. Due to its rising incidence worldwide and relatively poor prognosis, ICC remains a deadly disease [1]. ICC is characterized by abundant desmoplastic response [2]. The imaging features of ICC were well described; however, in addition to diagnosis the radiography also played an important role in patient prognostication and management [3]. Due to mucinous degeneration present in the center, the tumor rarely manifests calcification [4]. Although certain immunohistochemistry features are considered suggestive, definite histopathological identification of ICC remains difficult, and ICC ultimately remains a diagnosis of exclusion [5]. To get the prognostic biomarkers with high sensitivity and specificity, further immunohistochemical staining is necessary for differential diagnosis. It is important to develop new and specific biomarkers to aid diagnosis in early-stage ICC, and subsequently help guide the stratification of patients.

Sal-like protein 4 (SALL4), a stem cell marker expressed in fetal livers and various malignancies, is a zinc finger transcription factor that plays a role in maintaining self-renewal in embryonic stem cells and has been used as a marker of germ cell tumor [6], AFP-producing gastric carcinoma [7], and aggressive hepatocellular carcinoma (HCC) [8]. It was reported that the expression of SALL4 was upregulated in >50% of the HCCs [9]. Several recent studies have shown that SALL4 may play a critical role in carcinogenesis of HCC and implicate a more aggressive behavior [10–12], suggesting that SALL4 is a novel HCC marker, and a gene involved in embryogenesis and organogenesis.

Despite the significant difference of the therapeutic strategy for ICC and HCC, their histological appearance may be very similar. We now report that SALL4 expression occurs in ICC, and that association between SALL4 expression and the expression of Ki67, CA199, AFP, GGT, and P53 in ICC cases evaluated by immunohistochemical staining, and that the relationship between SALL4 expression and overall survival of patients in ICC.

## RESULTS

### High SALL4 expression was associated with metastasis and invasion in ICC

**Figure 1:**
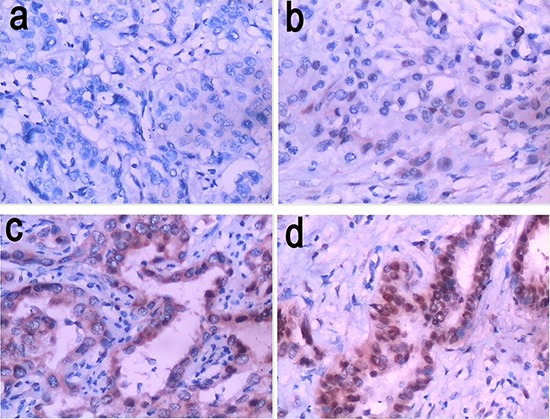
Representative images of SALL4 immunostaining (200× magnification) **a.** SALL4-negative expression (−); **b.** weak SALL4-positive expression (+); **c.** moderate SALL4-positive expression (++); **d.** strong SALL4-positive expression (+++).

As demonstrated in Figure 1, immunohistochemical data showed that the SALL4 protein was expressed mainly in the cytoplasm (brown). The SALL4 positive immunoreactivity was detected in a total of 102 ICC cases (58%), and non-reactivity was validated on tissue sections from 73 cases (42%), whereas non-reactivity was observed on total 28 adjacent non-tumor tissues. Moreover, the relationship between SALL4 protein expression and clinicopathological factors in ICC cases are shown in Table 1. The frequency of lymph node metastasis was significantly higher in SALL4-positive cases than in SALL4-negative cases (*P* = 0.0460). SALL4 expression was significantly correlated with vascular invasion and nerve invasion (*P* < 0.0001). In addition, we found no correlation between the expression of SALL4 and any other of the clinicopathological characteristics, including age, sex, tumor size, tumor locality, histologic grade, HBV infection, and clinical stage in ICC cases.

**Table 1:** Association between SALL4 expression and clinicopathologic characteristics in ICC patient

Variables	SALL4 expression		
	Positive (score = 1–4) *n* = 102 (%)	Negative (score = 0) *n* = 73 (%)	*P*
Age	53.52 ± 11.23	56.61 ± 13.26	0.853
Sex (%)			
Male	57(56)	36(49)	0.39
Female	45(44)	37(51)	
Tumor focality (%)			
Solitary	89(87)	64(88)	0.934
Multiple	13(13)	9(12)	
Tumor size (cm)	4.97 ± 2.32	6.27 ± 3.56	0.373
Histologic grade (%)			
Well differentiated	7(7)	6(8)	0.104
Moderately differentiated	77(75)	43(59)	
Poor differentiated	18 (18)	24(33)	
Nodal metastasis (%)			
Present	25(25)	9(12)	0.046
Absent	77(75)	64(88)	
Vascular invasion (%)			
Present	62(61)	6(8)	<0.0001
Absent	40(39)	67(92)	
Nerve invasion (%)			
Present	47(46)	6(8)	<0.0001
Absent	55(54)	67(92)	
HBV infection (%)			
Present	11(11)	11(15)	0.399
Absent	91(89)	62(85)	
Clinical T stage (%)			0.651
T1–2	58(57)	44(60)	
T3–4	44(43)	29(40)	

### SALL4 expression was correlated with Ki67 and CA199 levels in ICC cases

The relationship between SALL4 protein expression and the expression of Ki67, CA199, AFP, GGT, and P53 in ICC cases are shown in Table 2, the SALL4 expression was significantly correlated with the Ki67 and CA199 expression (both *P* < 0.0001). We found no correlation between the expression of SALL4 and any other of immunohistochemical findings, including AFP, GGT, and P53 in ICC cases.

**Table 2:** Association between the expression of SALL4 and other markers in ICC

Variables	SALL4 expression		
	Positive (score = 1–4) *n* = 102 (%)	Negative (score = 0) *n* = 73 (%)	*P*
Ki67 expression			
Positive	96(94)	17(23)	<0.0001
Negative	6(6)	56(77)	
CA199 expression			
Positive	97(95)	4(5)	<0.0001
Negative	5(5)	69(95)	
AFP expression			0.199
Positive	63(62)	38(52)	
Negative	39(38)	35(48)	
GGT expression			
Positive	59(75)	42(59)	0.967
Negative	43(18)	31(33)	
P53 expression			
Positive	68(67)	41(56)	0.157
Negative	34(33)	32(44)	

### High SALL4 expression predicted poor survival in patients with ICC

**Figure 2:**
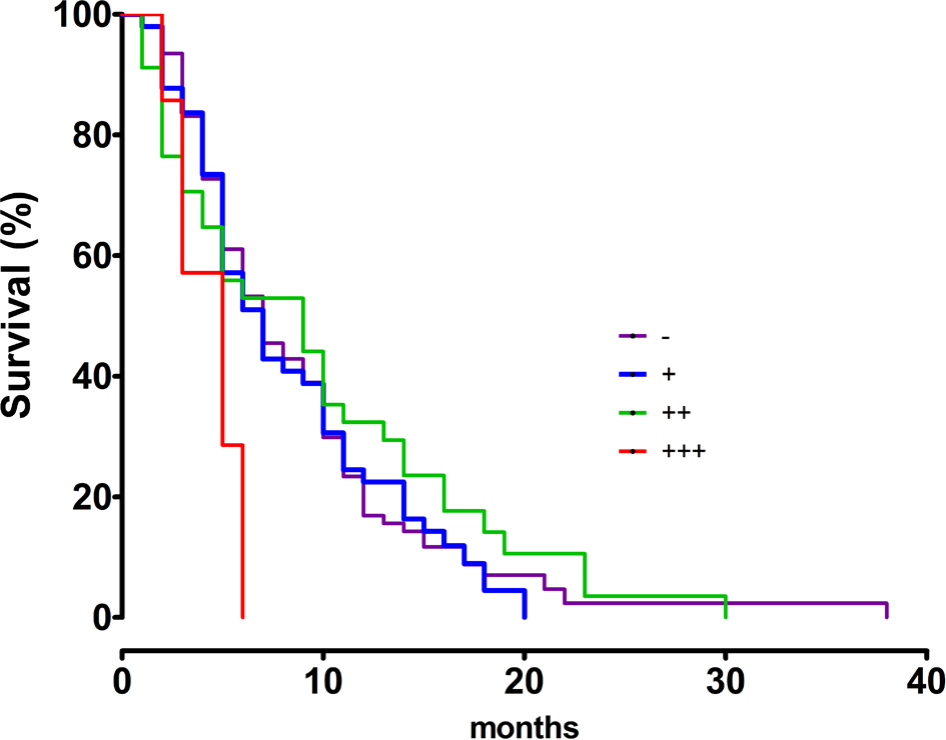
The overall survival curves for SALL4-negative (−) (*n* = 73), weak SALL4-positive cases (+) (*n* = 49), moderate SALL4-positive cases (++) (*n* = 46) and strong SALL4-positive cases (+++) (*n* = 7) in ICC

We carried out overall survival analysis (Kaplan-Meier analysis) for these 175 ICC patients. The analysis showed ICC patients with strong SALL4-positive levels (+++) had poorer prognosis than patients with moderate SALL4-positive levels (++) (*P* = 0.0471), weak SALL4-positive levels (+) (*P* = 0.014), and SALL4-negative expression (−) (*P* = 0.0055) (Figure 2). The median survival period of SALL4-negative patients, weak and moderate SALL4-positive cases were 7 months, 7 months and 9 months, respectively, whereas the median survival period of patients with strong SALL4-positive expression was only 5 months.

### SALL4 knockdown inhibited proliferation, migration and invasion of ICC cells

**Figure 3:**
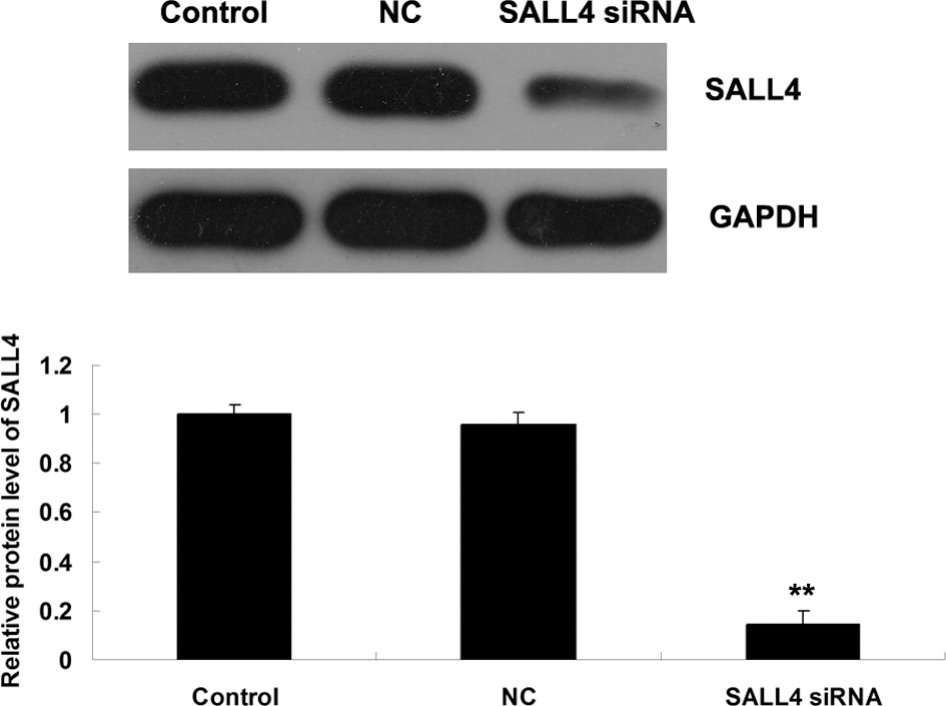
Western blot was conducted to determine the protein level of SALL4 in ICC-9810 cells transfected with SALL4 siRNA or non-specific siRNA as negative control (NC). GAPDH was used as loading control. Non-transfected ICC-9810 cells were used as Control. **P < 0.01 vs. Control

**Figure 4:**
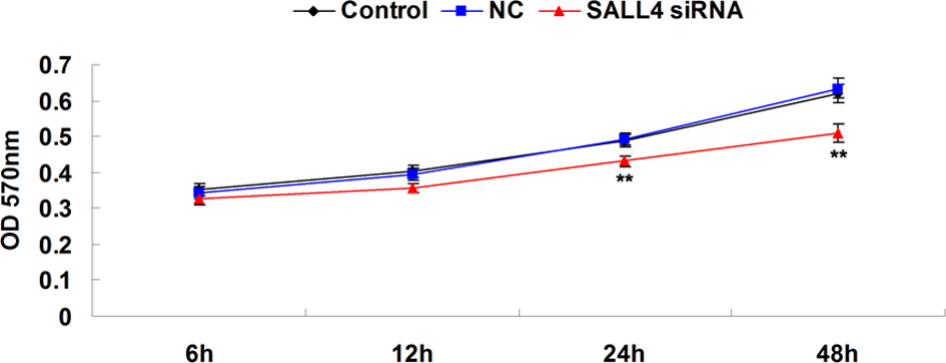
MTT assay was conducted to determine the cell proliferating capacity of ICC-9810 cells transfected with SALL4 siRNA or non-specific siRNA as negative control (NC). Non-transfected ICC-9810 cells were used as Control. **P < 0.01 vs. Control

**Figure 5:**
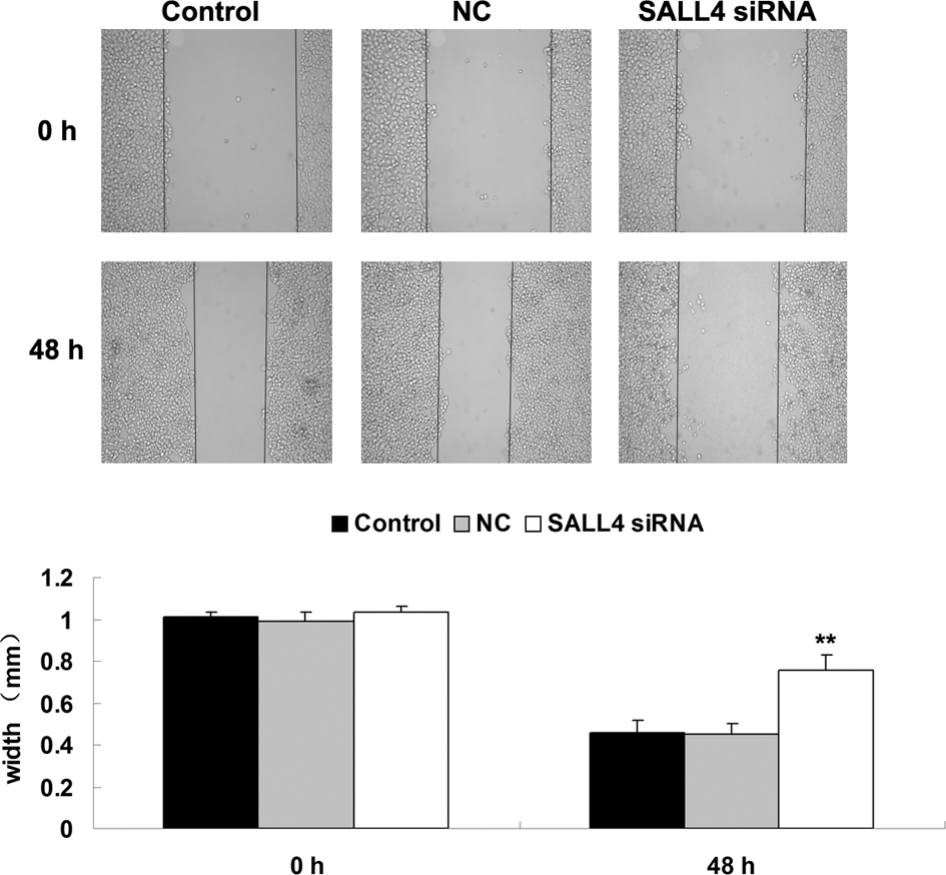
Wound healing assay was conducted to determine the cell migratory capacity of ICC-9810 cells transfected with SALL4 siRNA or non-specific siRNA as negative control (NC). Non-transfected ICC-9810 cells were used as Control. **P < 0.01 vs. Control

**Figure 6:**
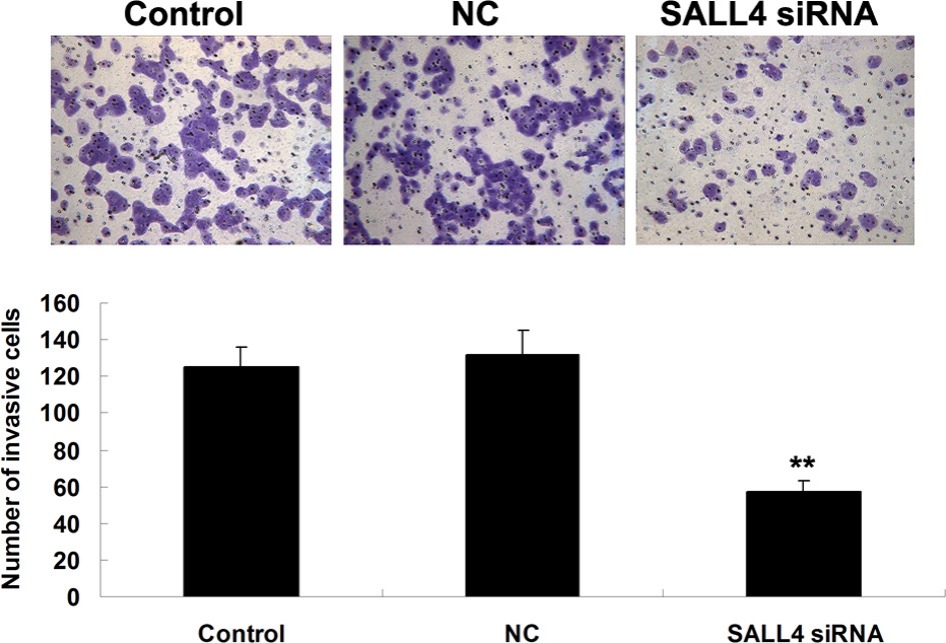
Transwell assay was conducted to determine the cell invasive capacity of ICC-9810 cells transfected with SALL4 siRNA or non-specific siRNA as negative control (NC). Non-transfected ICC-9810 cells were used as Control. **P < 0.01 vs. Control

We further investigated the role of SALL4 in ICC-9810 *in vitro*. We transfected ICC-9810 cells with SALL4-specific siRNA, and found that the protein expression level of SALL4 was notably reduced in SALL4 siRNA transfected cells, compared to the controls (Figure 3). After that, we compared the cell proliferation, migration and invasion between the control cells and SALL4 siRNA transfected cells. As shown in Figures 4–6, the cell proliferation, migration and invasion were all suppressed in ICC-9810 cells transfected with SALL4 siRNA, when compared to the control. These findings suggest that SALL4 plays an oncogenic role in ICC, and siRNA-induced SALL4 downregulation can inhibit malignant phenotypes of ICC cells.

**Figure 7:**
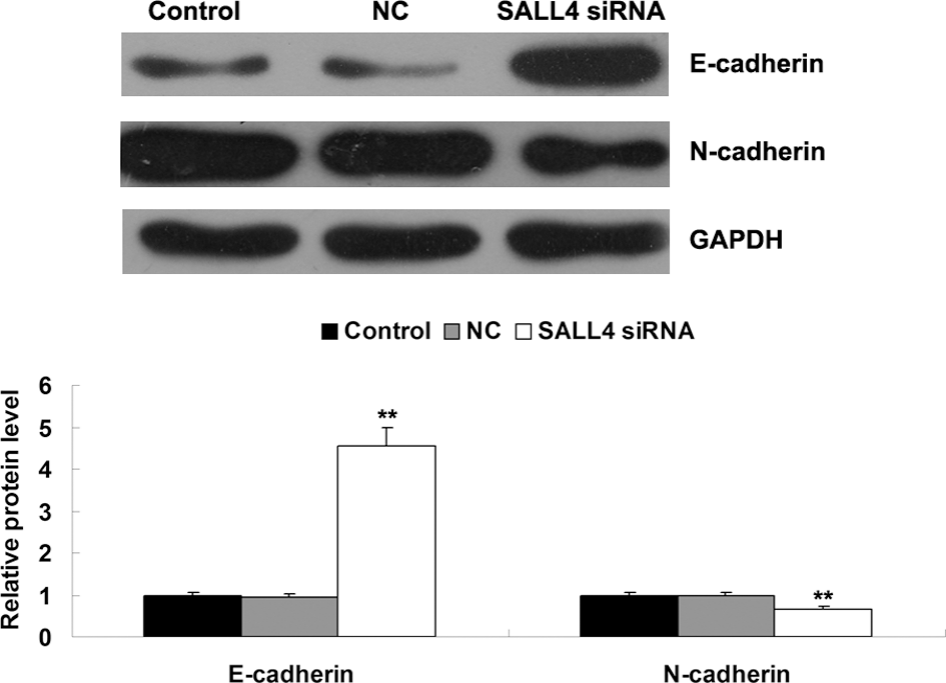
Western blot was conducted to determine the protein levels of E-cadherin and N-cadherin in ICC-9810 cells transfected with SALL4 siRNA or non-specific siRNA as negative control (NC). GAPDH was used as loading control. Non-transfected ICC-9810 cells were used as Control. **P < 0.01 vs. Control

The SALL4 expression was significantly correlated with vascular invasion and nerve invasion, suggesting that SALL4 may play a role in ICC metastasis. Therefore, we further determined the expression of some key genes involved in epithelial-mesenchymal transition (EMT), which is tightly associated with cancer metastasis [5]. As shown in Figure 7, the expression of E-cadherin was notably upregulated, but the N-cadherin protein level was significantly reduced after knockdown of SALL4 in ICC-9810 cells, suggesting that siRNA-induced SALL4 downregulation shows an inhibitory effect on ICC metastasis.

## DISCUSSION

The oncofetal protein SALL4 has been shown to play an important role in the extensive network of heterogeneous cellular pathways underlying hepatocarcinogenesis, suggesting that blockade the oncogenic role of SALL4 confers therapeutic potential in SALL4-positive HCC [13]. Recently, it was reported that SALL4 acted as a highly sensitive and specific marker for primary and metastatic gonadal and extragonadal yolk sac tumor [14]. Yolk sac tumor and HCC share similar histologic, serologic, and immunohistochemical features. Some previous studies reported that SALL4 expression was lack of in Western HCC patients, which SALL4 immunoreactivity was seen only in 3 of 236 cases (1.3%) in a large Western HCC cohort [15]. However, in line with the another study on Asian patients with HCC [16], we found at least focal SALL4 nuclear expression in up to 58.2% (102/175) of ICC cases, while in none of total 28 adjacent cancer tissues, suggesting that ICC and HCC histological appearance are very similar and a significant racial difference.

In this study, we found that the SALL4 expression in ICC was significantly correlated with vascular invasion and nerve invasion, and lymph node metastasis. Although a previous study showed that SALL4 expression status was not associated with the HCC tumor-node-metastasis and Barcelona Clinic Liver Cancer stages in either the Singapore cohort or the Hong Kong cohort [13], SALL4 was also reported to regulate cell proliferation through regulating the cyclin D1 and cyclin D2 expressions and promote the expression of genes involved in EMT that occurs in invasion and metastasis of cancer cells [9]. Moreover, we also found that the SALL4 expression was significantly correlated with Ki67 expression and CA199 expression. Upregulations of Ki67 and CA199 are the markers for increased proliferation of cancer cells. Serial reports have shown that SALL4 is required for cell proliferation and maintenance of pluripotency in several types of stem cells, as well as in malignantly transformed stem cells (e.g. leukemia and breast cancer) [17–19]. Knocking down SALL4 caused a decrease in cell viability, an increase in apoptosis, and a decrease in tumorigenicity of HCC cells [13]. Overexpression of either SALL4 isoform in hematopoietic stem cells or progenitors impairs hematopoietic colony formation and expansion *in vitro* [20]. Thus, it is reasonable that upregulation of SALL4 expression increased proliferation of cancer cells and enhanced the capacity of invasion and metastasis of cancer cells, subsequently promoted vascular invasion and nerve invasion in ICC.

In this study, overall survival analysis showed that patients with a high level of SALL4 expression had a worse prognosis than patients with a negative and low level of SALL4 expression in ICC. The blood copy number of SALL4 in colorectal cancer patients was significantly higher than healthy controls, which was inversely associated with the depth of tumor invasion and the high grade of tumor differentiation [21]. A combination of palate, lung, and nasal epithelium carcinoma-associated protein (PLUNC), hepatocyte paraffin 1, and SALL4 were reliable prognostic indicators in hepatoid adenocarcinoma of the stomach [7]. In addition, low level of SALL4 expression was inversely correlated with metastasis of esophageal squamous cell carcinoma cells into the lymph nodes [22]. Taken together, SALL4 is an indicator of aggressiveness and poor prognosis in ICC.

The SALL4 expression was significantly correlated with the Ki67 expression, vascular invasion and nerve invasion, suggesting that SALL4 may play a role in ICC cell proliferation, migration and invasion. To verify this speculation, we applied SALL4 siRNA to knock down the expression of SALL4 in ICC cells, and found that siRNA-induced SALL4 inhibition significantly suppressed ICC cell proliferation, migration and invasion, accompanied with increased E-cadherin expression as well as downregulation of N-cadherin, indicating an inhibition of EMT. Similar findings were also reported in ALK-positive anaplastic large cell lymphoma (ALK+ALCL), gastric cancer, lung cancer, HCC, breast cancer, endometrial cancer and esophageal squamous cell carcinoma [11, 22–27]. For instance, knockdown of SALL4 resulted in apoptosis and cell-cycle arrest in ALK+ALCL cells [23]. siRNA-mediated SALL4 inhibition led to inhibition of lung cancer cell proliferation that was induced by cell cycle arrest at the G1/early S phase [25]. These findings suggest that the suppressive effect of SALL4 downregulation on ICC cell proliferation may be also due to cell apoptosis and/or cell-cycle arrest. Zhang et al. found that the expression of SALL4 was highly correlated with lymph node metastasis of gastric cancer, and knockdown of SALL4 by siRNA led to the decreased proliferation and migration of human gastric cancer cells. Besides, they also found that the expression level of SALL4 was associated with EMT in gastric cancer cells [24]. Itou et al. showed that SALL4 could inhibit intercellular adhesion and maintain cell motility after cell-cell interaction and cell division, resulting in the dispersed phenotype in basal-like breast cancer [26].

To our knowledge, this is the first study reporting that SALL4 plays an oncogenic role in ICC. According to our data, we suggest that SALL4 may become a potential target for the treatment of ICC growth and metastasis.

## MATERIALS AND METHODS

### Tissue samples

This study was approved by the legislation and ethical boards of Central South University, Changsha, China. All subjects or their caregivers have written informed consent. A total of 175 ICC tissue sections and 28 adjacent tissues of cancer were collected from the Department of Hepatobiliary and Pancreatic Surgery, Third Xiangya Hospital of Central South University. The clinicopathologic characteristics of the ICC samples including tumor size, location, histologic grade, clinical stage, HBV infection, lymph node metastasis, vascular invasion, and nerve invasion were recorded and summarized in Table 3. Samples were classified according to the criteria of American Joint Committee on Cancer stage Staging Manual, Seventh Edition (2010) for ICC, and all tissue sections were confirmed by the original histopathological and clinical diagnosis. No preoperative chemotherapy, radiotherapy, or embolization was used in these patients. All these samples were formalin fixed and paraffin-embedded.

**Table 3:** Clinicopathologic characteristics of ICC patients

	ICC patients (*n* = 175)
Age	
Mean ± SD	55 ± 13.58
Sex%	
Male	53(93/175)
Female	47(82/175)
Tumor focality (%)	
Solitary	87(153/175)
Multiple	13(22/175)
Tumor size (cm)	
Mean ± SD	5.08 ± 3.27
Histologic grade (%)	
Well differentiated	7(13/175)
Moderately differentiated	68(120/175)
Poor differentiated	25(42/175)
Nodal metastasis (%)	
Present	19(34/175)
Absent	81(141/175)
Vascular invasion (%)	
Present	39(68/175)
Absent	61(107/175)
Nerve invasion (%)	
Present	30(53/175)
Absent	70(122/175)
HBV infection (%)	
Present	12(21/175)
Absent	88(153/175)
Clinical T stage (%)	
T1	16(28/175)
T2	42(74/175)
T3	30(52/175)
T4	12(21/175)

### Immunohistochemical staining assay

The expression of SALL4 was evaluated by using immunohistochemical staining. Four μm sections were deparaffinized and subjected to heat-induced antigen retrieval using citrate buffer for 22 minutes using a microwave oven. Then the sections were incubated with antibodies against SALL4 (1:100, Sigma Aldrich, St. Louis, MO, USA). Subsequently, the sections were incubated with secondary antibody for 60 minutes at room temperature. The reaction was developed using substrate diaminobenzidine (DAB) and counterstained with hematoxylin, and visualized via optical microscopy. For evaluating the expression of SALL4 in ICC tissues, extent, intensity, and pattern of nuclear expression were assessed in each spot. All tissue sections were analyzed and scored independently by three experienced pathologists. The scoring system was as follows: the percentage of positively staining cells was graded as - (no staining, SALL4-negative), + (>0 and ≤ 25% of cells positive, weak SALL4-positive), ++ (>25 and ≤75% of cells positive, moderate SALL4-positive), and +++ (>75% of cells positive, strong SALL4-positive). Besides, immunohistochemical staining for Ki67 (1:200; Abcam, Cambridge, UK), AFP (1:150; Abcam), GGT (1:200; Santa Cruz, Dallas, Texas) and P53 (1:100; Sigma Aldrich) was performed in a similar way as described above, respectively. Extent and intensity of expression of both markers were scored using the same approach as for SALL4.

### Cell culture

The human ICC-9810 cell line was purchased from the Institute of Cell Biology at the Chinese Academy of Sciences (Shanghai, China). ICC-9810 cells were cultured in DMEM added with 10% fetal bovine serum (FBS, Life Technologies) at 37°C in a humidified incubator containing 5% CO_2_.

### Transfection

Cells were cultured into 70% confluence. Lipofectamine 2000 (Life Technologies) was used to perform transfection according to the manufacture’s instruction. Briefly, SALL4 siRNA (Santa Cruz), non-specific siRNA (Santa Cruz), or Lipofectamine 2000 was diluted with serum-free medium, respectively. The diluted Lipofectamine 2000 was then added into the diluted siRNA, and incubated for 20 minutes at room temperature, and then added into the cell medium. Then, cells were incubated at 37°C, 5% CO_2_ for 6 hours. After that, the medium in each well was replaced by the normal serum-containing medium, and cultured for 24 hours before the following assays.

### Western blot

Cells were solubilized in cold RIPA lysis buffer. Proteins were separated with 12% SDS-PAGE, and transferred onto a polyvinylidene difluoride (PVDF) membrane. The membrane was incubated with TBST containing 5% skimmed milk at 37°C for 2 hours. Then, the membrane was incubated with mouse anti-SALL4 (1:100; Sigma Aldrich), mouse anti-N-cadherin (1:100; Sigma Aldrich), mouse anti-E-cadherin (1:400; Sigma Aldrich), and mouse anti-GAPDH primary antibodies (1:200; Sigma Aldrich), respectively, at room temperature for 2 hours. After being washed by PBST for 4 times with 10 minutes each time, the membrane was incubated with the goat anti-mouse secondary antibodies (1:5000; Sigma Aldrich) at 4°C overnight. After being washed by PBST for 4 times with 10 minutes each time, ECL kit (Pierce Chemical, Rockford, IL, USA) was used to perform chemiluminent detection. Image-Pro plus software 6.0 was used to analyze the relative protein expression, represented as the density ratio versus GAPDH.

### Cell proliferation assay

MTT assay was used to measure cell proliferation. At 72-hour post-transfection, 100 μl cell suspension (5000 cells/ml) was seeded into 96-well plate, and incubated at 37°C with 5% CO_2_ for 6, 12, 24, and 48 hours, respectively. For MTT assay, the transfection medium in each well was replaced by 100 μl of fresh serum-free medium with 0.5 g/l MTT. After incubation at 37°C for 4 h, the MTT medium was removed by aspiration and 50 μl of DMSO was added to each well. After reacting for 10 minutes at room temperature, formazan production was detected by measurement of the optical density (OD) at 570 nm using an ELX-800 type ELISA reader (Bio-Tek, VT, USA).

### Wound healing assay

Wound healing assay was performed to evaluate the cell migratory capacity. In brief, ICC-9810 cells were cultured to full confluence. Wounds of approximately 1 mm width were created with a plastic scriber, and cells were washed. After cultured for 48 hours, cells were fixed and observed under a microscope.

### Cell invasion assay

For cell invasion assay, 24-well transwell chambers (Chemicon, CA, USA) that has a layer of matrix gel were used. For each group, cell suspension (500000 cells/ml) was prepared in serum free DMEM, and 500 μl of DMEM with 10% FBS was added into the lower chamber, and 300 μl of cell suspension was added into the upper chamber. After incubation at 37°C with 5% CO_2_ for 24 hours, those non-invading cells and the matrix gel were removed, and cells through the membrane were stained for 20 minutes, and then rinsed by water, and dried in air. Five fields were randomly selected under the microscope (Nikon), and the stained cell number was counted.

### Statistical analysis

SPSS software (version 16.0) and Graphpad Prism 5 were used for statistical analyses. Continuous variables were expressed as mean ± SD or median ± range, whichever was applicable; categorical variables were expressed as percentages. Associations between SALL4 expression and clinicopathologic characteristics in ICC were assessed using the χ^2^ test, Fisher exact test, and Student *t* test. For *in-vitro* experiments, statistical analysis of differences was performed by one-way analysis of variance (ANOVA). The survival data was analyzed by Kaplan-Meier curve and the difference among different groups was detected by log-rank test. *P* < 0.05 was considered to be statistically significant.
